# Preliminary in-vitro antibacterial evaluation of a novel formula of calcium phosphate nanoparticle–chlorhexidine intracanal medicament using confocal laser scanning microscopy

**DOI:** 10.1186/s12903-026-08428-x

**Published:** 2026-05-07

**Authors:** Mohamed Salah eldin Omran, Sarah Hossam Fahmy, Nawar Naguib Nawar, Abeer A ElHakim ElGendy, Edgar Schäfer

**Affiliations:** 1 Faculty of Dentistry, Ain Shams University, Cairo, Egypt; 2 Faculty of Dentistry, The British University in Egypt, Cairo, Egypt; 3 Central Interdisciplinary Ambulance in the School of Dentistry, University of Münster, Münster, Germany

**Keywords:** Calcium phosphate nanoparticles, Confocal laser scanning microscopy, *E. faecalis* biofilm, Intracanal medicament, Root canal disinfection

## Abstract

**Background:**

Bacterial infection in root canals often persist despite thorough chemomechanical preparation. Conventional irrigants and medicaments reduce biofilms but have been accused of weakening dentin. Amorphous calcium phosphate nanoparticles showed promising results in enhancing dentin hardness, but the formula’s anticmicrobial effect is yet to be evaluated in endodontic contexts. The aim of this study was to evaluate the feasibility of using 20% amorphous calcium phosphate nanoparticles loaded in chlorhexidine as an antibacterial intracanal medicament in endodontics.

**Methods:**

Forty four root dentin discs were prepared from human extracted teeth, sterilized and inoculated with *E. faecalis* to establish a 3-week-old standard monospecies bacterial biofilm model. After confirming biofilm establishment, forty specimens were randomly divided into four groups (*n* = 10) according to the medicament used: 20% amorphous calcium phosphate nanoparticles loaded in 2% chlorhexidine (NACP + CHX), 2% chlorhexidine (CHX), calcium hydroxide (Ca(OH)_2_), and a positive control group. After incubation, the specimens were stained and evaluated under the confocal laser scanning microscope (CLSM). Images were analyzed with Zen imaging software to quantify the percentage of live/dead bacterial cells. For multiple group comparisons, a one-way ANOVA was employed, and for pairwise comparisons, the Tukey post hoc test was used. Statistical significance was determined by a p-value < 0.05.

**Results:**

All test groups showed a statistically significant bacterial reduction, ranging from 36.81% to 59.19% (*p* ≤ 0.001). CLSM analysis showed that NACP + CHX and CHX had the highest antibacterial effect without a significant difference between them (*p* ≈ 0.9). Ca(OH)_2_ demonstrated the least antibacterial effect amongst the test groups (*p* ≈ 0.01 × 10^-6^).

**Conclusion:**

The findings suggest that NACP + CHX when used as an intracanal medicament, demonstrates antibiofilm efficacy against E. faecalis biofilm.

**Supplementary Information:**

The online version contains supplementary material available at 10.1186/s12903-026-08428-x.

## Background

The current microbiological endodontic concepts emphasize that endodontic disease is essentially a biofilm-mediated infection [1]. Bacterial biofilm mode of growth is an adaptive process that allows bacteria to survive nutritional deprivation and antibacterial measures in treated root canals [2, 3]. Consequently, elimination or reduction of bacterial biofilms is a prerequisite for the success of endodontic treatment [4, 5]. However, clinical studies have demonstrated that even with meticulous chemomechanical disinfection and obturation procedures, bacterial biofilms are not always completely eradicated from the root canal system [5]. Root canal systems vary wildly [6], and this morphological variation hinders the accessibility to a degree that no root canal instrument to date could achieve shaping of the entire root canal system [7]. Although complete bacterial eradication from the root canal system is still unattainable, contemporary chemomechanical preparation significantly reduces microbial load and is associated with high clinical success rates; nevertheless, residual microorganisms may persist within anatomical complexities despite the use of irrigants and activation techniques [5, 8]. In this context, intracanal medicaments are advocated as an adjunct to prolong antibacterial contact and enhance overall disinfection by prolonging antibacterial contact and enhanced disinfection [4, 9, 10].

Subsequently, to disrupt bacterial biofilms and eradicate bacterial infections, several intracanal medicaments were developed. The most commonly used intracanal medicaments are calcium hydroxide (Ca(OH)_2_) and chlorhexidine (CHX), however, both agents have their limitations [11]. Ca(OH)_2_, although widely used, demonstrates reduced antibacterial effectiveness against established biofilms due to its low diffusibility and solubility [12], the buffering effect of dentin [11], and the protective extracellular matrix that characterizes mature biofilm structures [1, 11]. Likewise, CHX exhibits broad-spectrum antimicrobial activity, yet it may become inactivated by physiological salts [13], shows limited penetration into the deeper layers of biofilms [14], and does not consistently eliminate biofilm-embedded microorganisms. Consequently, residual bacteria may persist within dentinal tubules and anatomical irregularities [1]. Given these limitations, efforts have been directed toward the incorporation of nanoparticles as intracanal medicaments, owing to their broad-spectrum antimicrobial potential [15].

Due to their nanoscale dimensions, high surface-area-to-volume ratio, and surface charge characteristics, nanoparticles demonstrate enhanced interactions with bacterial membranes compared to their macroscale counterparts [15–17]. Calcium phosphate is a widely available and easy-to-manufacture material that has fallen out of interest in nanoparticle research due to its perceived simplicity [18]. However, recent studies have demonstrated the ability of the nanoparticles of amorphous calcium phosphate (NACP) to significantly enhance the radicular dentin microhardness [19–22]. This is particularly important in the ongoing debate about the impact of root canal shaping on the biomechanical properties of teeth [23–25], especially concerning whether the use of Ca(OH)_2_ negatively affects dentinal strength and compromises the clinical longevity of the tooth [26]. Thus, it may be worth revisiting calcium phosphate nanoparticles, viewing their simplicity as a potential strength if they can both strengthen dentin and exhibit effective antibacterial properties.

As for antibacterial efficiency, the NACP exhibited signs of an antibacterial effect, being more effective against Gram-positive species and having a synergistic effect with certain antibiotics including ampicillin, oxacillin, vancomycin, and clindamycin [18]. Such synergism allowed antibiotics that were previously deemed completely ineffective against certain bacterial strains to significantly suppress their growth. NACP was also shown to induce remineralization in both enamel and dentin, decrease biofilm lactic acid production, and increase the phosphate and calcium content of the biofilm [27, 28]. Given these findings, this study aims to provide a preliminary evaluation for a novel combination of NACP nanoparticles loaded with chlorhexidine, comparing its antibacterial efficacy to two commonly used intracanal medicaments: 2% chlorhexidine and the gold standard, Ca(OH)_2_.

While the clinical reality of endodontic infections is polymicrobial [29], the use of E. faecalis mono-species biofilms remains a well-established in vitro model for preliminary antimicrobial evaluation [4]. This simplified model of a benchmark organism offers reproducibility and high-throughput screening potential, serving as a valid “starting point” before moving to more complex multispecies systems [4]. To the best of our knowledge, this is the first study to evaluate such NACP/CHX formulation as an intracanal medicament using confocal laser scanning microscopy (CLSM) assessing the antibacterial efficacy against a mature biofilm. The null hypothesis for this study was that there would be no statistically significant difference in antibacterial efficacy between the tested groups.

## Methods

**Fig. 1:**
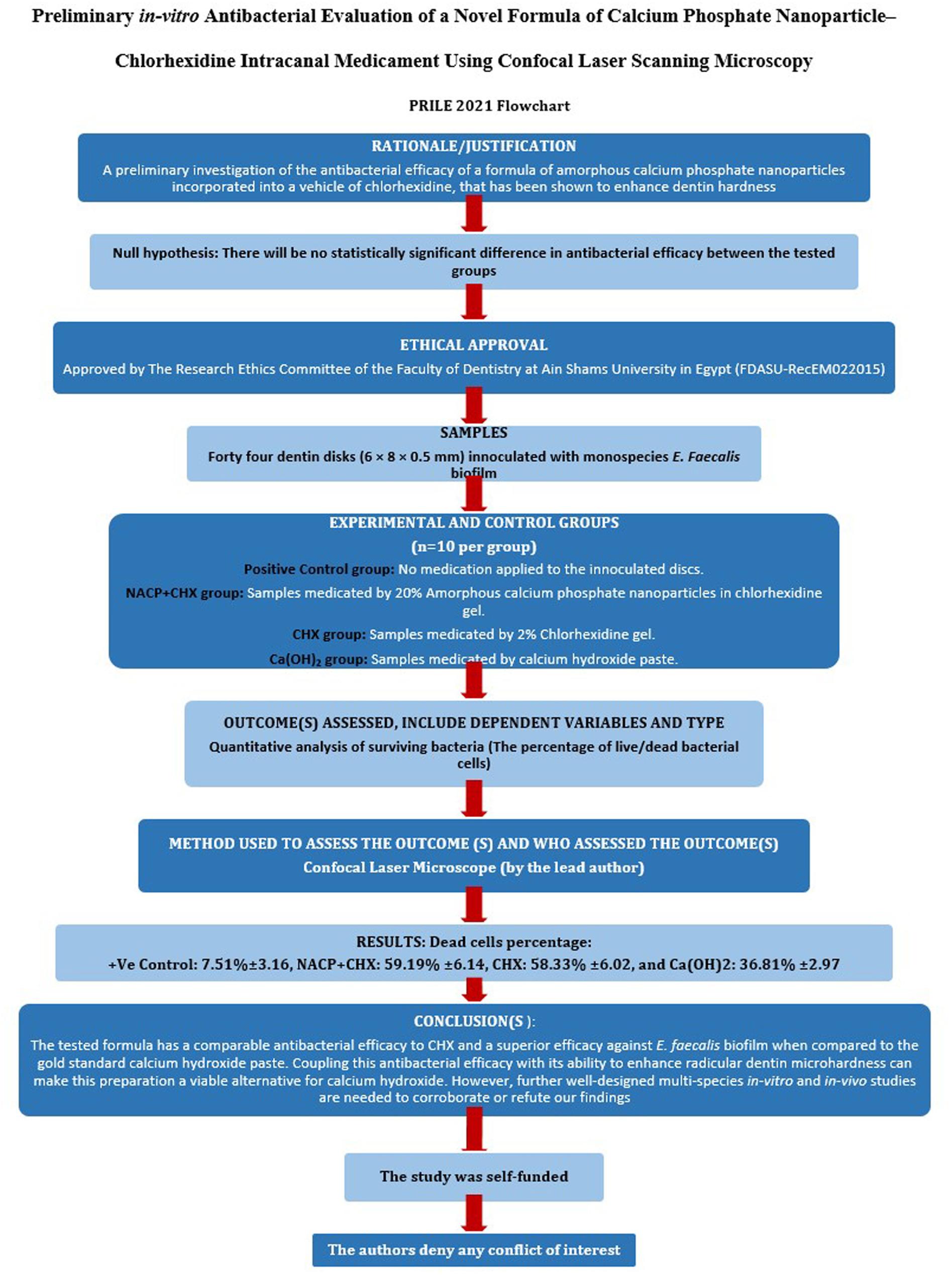
The PRILE flow-chart detailing the study design, methodology and findings

The manuscript of this laboratory study has been written according to Preferred Reporting Items for Laboratory studies in Endodontology (PRILE) 2021 guidelines [30]. A flowchart demonstrates the study design and findings accordingly (Fig. 1).

### Ethical considerations and sample size calculation

The study was approved by The Research Ethics Committee of the Faculty of Dentistry at Ain Shams University in Egypt (FDASU-RecEM022015), and all procedures involving human specimens were conducted in full accordance with relevant regulations, including the Declaration of Helsinki. The sample size calculation was done using G*Power 3.1 software (Heinrich-Heine-Universitat, Dusseldorf, Germany) for a fixed-effects one-way ANOVA (omnibus test). An effect size (f) of 0.25, representing a moderate effect according to Cohen’s criteria, was adopted based on variability reported in previous similar experimental models of de Almeida et al. [31]. With α = 0.05 and a statistical power of 0.90, the required total sample size for four groups was calculated as 40 specimens (10 per group). The anonymized teeth belonged to patients undergoing routine dental extractions, for reasons unrelated to the study, and who had provided informed consents for the use of their extracted teeth in scientific research rather than incineration.

### Dentin specimens preparation and sterilization

Forty four intact human mature permanent single-rooted teeth were collected from the oral and maxillofacial surgery department. Confirmation of single canal configuration was achieved through mesio-distal and bucco-lingual radiographs. Bone, calculus, and periodontal tissues were removed mechanically using an ultrasonic scaler before teeth were inspected under magnification to exclude fractures, cracks, or craze lines. Specimens were prepared according to a described protocol in a previous study [32]. A diamond disc was used to obtain the middle third of the root, by cutting away the coronal and the apical thirds. Size #35/0.06 ProFile rotary files (Dentsply Maillefer, Ballaigues, Switzerland) were used to prepare the canal lumen to a standardized size. Forty four dentin disks of 6 × 8 × 0.5 mm were obtained from the inner surface of the root halves, by sectioning the middle thirds buccolingually under constant cooling with distilled water using a sectioning saw (IsoMet 2000 Precision Saw; IsoMet, Buehler, IL).

Dentin sections were placed in an ultrasonic bath of 5.25% sodium hypochlorite and 17% EDTA for 4 min each to remove the smear layer, then rinsed with sterile water for 1 min. Each dentin section was placed in a separate 1.5 mL Eppendorf tube filled with brain heart infusion (BHI) broth, then autoclaved for 20 min at 121 °C and pressure of 15 PSI. Two discs were incubated for 24 h at 37 °C in BHI broth to ensure the absence of bacterial contamination (i.e. negative control) [32]. Surface polishing was not performed in order to preserve the biofilm architecture and avoid artificial modification of surface topography.

### Specimens inoculation with Enterococcus faecalis

The experiment involved cultivating a pure culture of *E*. *faecalis* (ATCC 29212), which was incubated anaerobically at 37 °C for 24 h in BHI broth. The resulting bacterial colonies were subsequently diluted in fresh BHI broth to achieve a turbidity equivalent to a 0.5 McFarland standard, corresponding to an optical density of 0.08–0.1 absorbance at 600 nm in a spectrophotometer. Each sterilized dentin disk was placed in a sterilized 12-well tissue culture plate (Nunc; Thermo Scientific, Darmstadt, Germany), with the pulpal side facing upward. A 3.0 mL suspension of *E*. *faecalis* (1 × 10^8^ CFU/mL) was added to each dentin disk, and the plate was incubated anaerobically at 37 °C for three weeks. The growth medium was changed every 72 h to maintain a consistent rate of microbial growth [32].

### Confirmation of biofilm formation

**Fig. 2:**
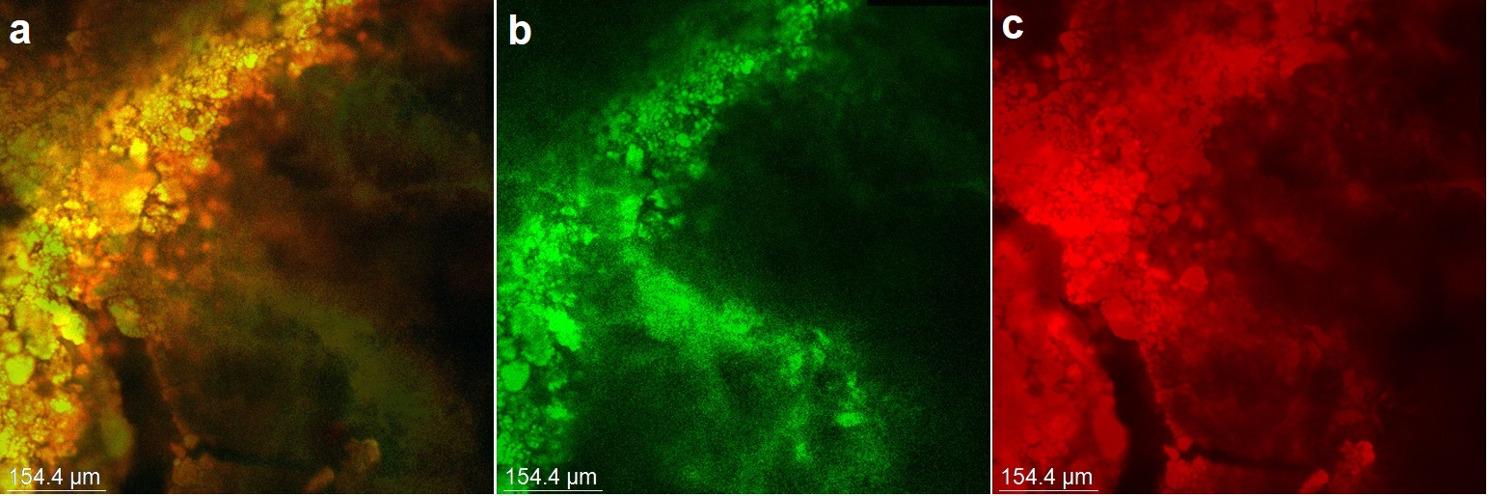
Confocal laser scanning micrograph (**a**): The green channel (**b**) shows live bacteria, and the red channel (**c**) shows dead bacteria

Two specimens were randomly selected and scanned under the confocal laser scanning microscope, before the application of any medicaments to confirm the formation of a mature *E. faecalis* biofilm (Fig. 2).

### Specimens classification

After incubation, the remaining 40 specimens were aseptically removed from the wells and rinsed gently with sterile phosphate-buffered saline for 1 min. The specimens were randomly assigned according to the type of medicament applied (10 samples per group) into four groups using computer-generated randomization (www.randome.org): A positive control group; a NACP + CHX group where the samples were medicated with 20% Amorphous calcium phosphate nanoparticles in chlorhexidine gel; then a CHX group where the samples were medicated with 2% Chlorhexidine gel (GLUCO-CHeX 2%, Cerkamed, Kwiatkowskiego, Poland), and the fourth group was Ca(OH)_2_ group where samples were medicated by an aquaeous Ca(OH)_2_ paste (Metapex, Meta Biomed, Cheongju, South Korea).

### Synthesis of NACP + CHX

NACP (Ca_3_[PO_4_]_2_) was synthesized through a spray-drying technique, as previously reported [33, 34]. Briefly, to produce NACP particles, dicalcium phosphate and calcium carbonate were dissolved in an acetic acid solution, resulting in a final ionic concentration of calcium and phosphate at 8 mmol/L and 5.333 mmol/L, respectively. The calcium/phosphate molar ratio of the solution was 1.5, which corresponded to that of ACP. Finally, the solution was sprayed into a heated chamber, and the resulting particles were collected using an electrostatic precipitator. The particles had a mean size of 106 nm; close to the 100 nm mean size described by Xu et al. [34]. 50 mL of gelatin solution were combined with 25 mL of chlorhexidine solution with a concentration of 0.4 mg/L, and 1 g of calcium phosphate nanoparticles in a 100 mL flask. The mixture was then stirred for one hour, to ensure uniform distribution of the nanoparticles within the chlorhexidine. The final pH was adjusted to a range of 6.5–7.5, by adding 0.1 N of sodium hydroxide solution [35], and the final concentration of CHX in the NACP + CHX mixture was adjusted to 2% to ensure comparability with the CHX control group.

### Medicaments application

Medicaments were applied to the specimens of the three experimental groups and laid in sterile centrifuge tubes. Anaerobic incubation was performed for 2 weeks at 37 °C and 100% humidity [36]. Each specimen was then washed with 5 mL of sterile saline to remove the corresponding test medicament. The positive control group received no medicaments before incubation, then it was exposed to sterile saline solution before being viewed under the confocal laser scanning microscope.

### Confocal laser scanning microscope (CLSM)

CLSM was used by a blinded assessor to directly visualize the live/dead bacteria in the four groups. As per Balto et al. the 4 corners of each dentin disk were scanned with a 2-µm step size at a resolution of 1024 × 1024 pixels [32]. Root specimens were stained utilizing Acridine orange/Propidium iodide Cell Viability Kit (Logos Biosystems, Inc, Gyeonggi-do, SK) for 15 min in a dark room, according to the manufacturer’s instructions. Root sections were then transferred to glass coverslips and covered with immersion oil before imaging [37]. A Zeiss LSM 710 confocal microscope (Carl Zeiss, Oberkochen, Germany) set at excitation/emission wavelengths of 502/525 nm for acridine orange and 490/635 nm for propidium iodide to inspect the specimens. To minimize artifacts, laser intensity and gain settings were kept constant for all groups. Acridine orange stains living cells producing green fluorescence, while Propidium iodide stains dead cells producing red fluorescence. Sequential frame scan mode was utilized to avoid crosstalk. Specimens’ examination was performed using ×40 magnification oil immersion objective with an aperture of 1.4 & confocal pinhole of 88 Mm for channel 1 and 164 Mm for channel 2. CLSM images were acquired by the software Zen 2012 blue edition at a resolution of 1024 × 1024 pixels.

### CLSM analysis

Image stacks were visualized using the CLSM browser. The initial fluorescence datasets, corresponding to green and red emission channels, were separated into individual components and stored as grayscale images. Voxel dimensions were calibrated to accurately define spatial resolution. Quantification of total fluorescence per optical section was achieved by summing the voxel intensities from both grayscale channels. For viability assessment, two-dimensional analysis of CLSM images was conducted using the Zen 2012 Blue Edition software, enabling quantitative characterization of the total biofilm as well as its subpopulations distinguished by green (viable cells) and red (non-viable cells) fluorescence signals (Fig. 2). The software calculated the relative biovolumes of live and dead cells by analyzing fluorescence intensities, thereby providing insights into bacterial viability and membrane integrity. The proportion of dead biovolume was determined by dividing the red fluorescence intensity by the total fluorescence intensity and expressing the result as a percentage [38].

### Statistical analysis

The data obtained from the experiments were analyzed statistically using SPSS version 20 (IBM Corp., Armonk, NY). Numerical data were tested for normality by checking the distribution, calculating the mean and median values, and using the Smirnov, Kolmogorov-Smirnov, and Shapiro-Wilk tests. As data were distributed normally, means and standard deviations were calculated. For multiple group comparisons, a one-way ANOVA was employed, and for pairwise comparisons, the Tukey post hoc test was used. Statistical significance was determined by a *p* < 0.05.

## Results

**Fig. 3:**
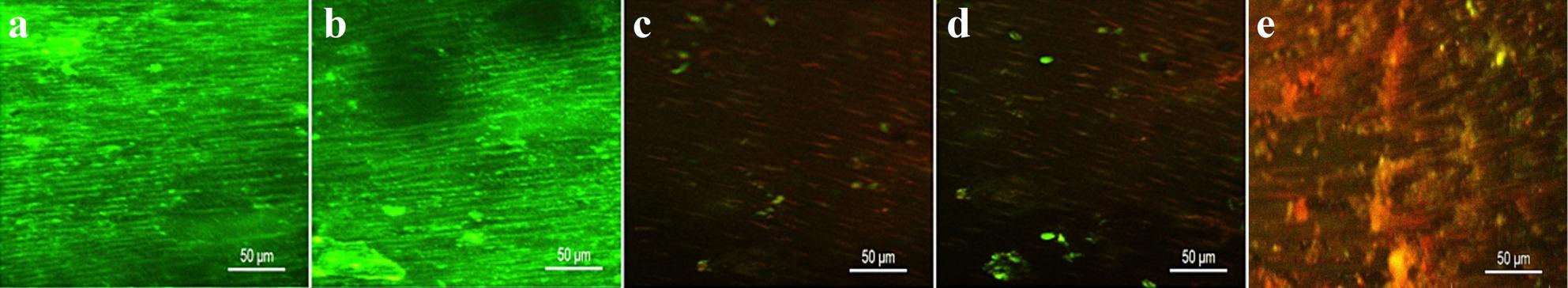
CLSM 2D images demonstrating live and dead bacteria within dentinal tubules. **a** Biofilm formation confirmation sample, (**b**) positive control group, (**c**) NACP + CHX group, (**d**) CHX group, and (**e**) Ca(OH)₂ group. Red fluorescence indicates dead bacteria, whereas green fluorescence indicates live bacteria. Images represent standardized fields acquired under identical magnification and imaging parameters. Scale bars = XX µm

**Fig. 4:**
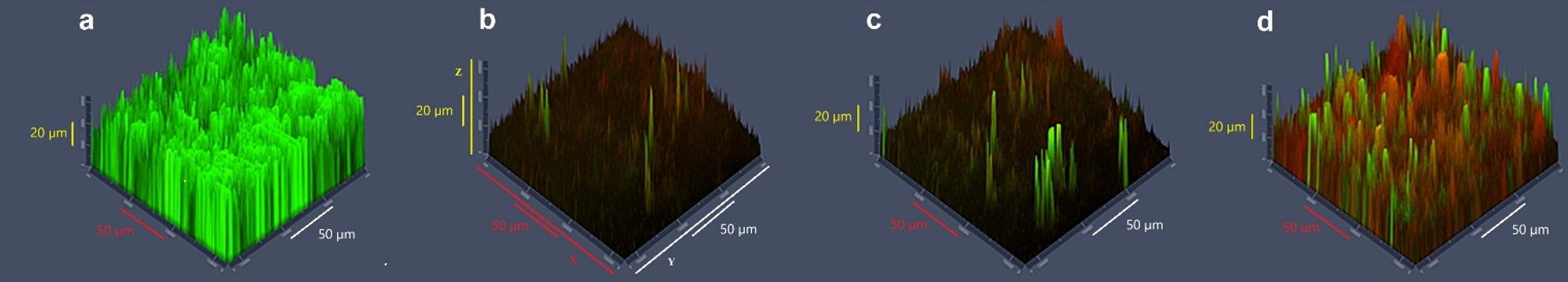
CLSM 2.5D reconstruction of live and dead bacteria within dentinal tubules. Green channel represents live bacteria and red channel represents dead bacteria. **a** Positive control group, (**b**) NACP + CHX group, (**c**) CHX group, and (**d**) Ca(OH)₂ group. The X- and Y-axes represent a 50 μm × 50 μm field of view, while the Z-axis represents a 20 μm depth. Color-coded scale bars are shown in each panel (X-axis: red; Y-axis: white; Z-axis: yellow). High peaks indicate areas of greater biofilm biomass or bacterial clustering, whereas lower regions reflect reduced biomass or structural irregularities

Mean percentages and standard deviation (SD) of dead cells are presented in Table-1, and a visual demonstration of the results is shown in Figs. 3 and 4. The highest mean percentage of dead cells in *E. faecalis* biofilm was recorded with Calcium phosphate nanoparticles in chlorhexidine (59.19% ±6.14), followed by Chlorhexidine (58.33% ±6.02). There was no statistically significant difference between both values, however, both values were statistically significantly higher than the Ca(OH)_2_ group (36.81% ±2.97). Finally, the lowest mean percentage of dead cells was recorded for the Control group (7.51% ±3.16). The 2D images in Fig. 3 demonstrate surface-level interactions of the biofilm in different groups, showcasing the distribution of live (green) and dead (red) bacteria. While the 2.5D images (Fig. 4) represent a pseudo three dimensional perspective, emphasizing the spatial distribution of live (green) and dead (red) bacteria across biofilm layers. One-way ANOVA demonstrated a statistically significant difference among groups (F(3,36) = 269.81, *p* < 0.001). The effect size was large (η² = 0.957), indicating that group allocation accounted for approximately 95.7% of the variance in percentage of dead cells. Post hoc analysis (Table 2) using Tukey’s HSD test further elucidated these differences. The NACP + CHX group (mean = 59.18%, SD = 6.14%) and the CHX group (mean = 58.62%, SD = 5.74%) exhibited the highest percentages of dead cells, with no statistically significant difference between them (*p* = 0.9934). Both NACP + CHX and CHX groups showed significantly higher dead cell percentages compared to the Ca(OH)₂ group (mean = 36.81%, SD = 2.97%) (*p* < 0.001 for both comparisons). The Ca(OH)₂ group, in turn, demonstrated a significantly higher percentage of dead cells than the Control group (mean = 7.06%, SD = 3.16%) (*p* < 0.001). The Control group had the lowest percentage of dead cells, significantly lower than all other experimental groups (*p* < 0.001 for all comparisons).

**Table 1:** Mean (%) and standard deviation (SD) of dead cells after two weeks of medicaments’ application

Group	Dead Cells count (%)
Positive Control	7.51% ±3.16^c^
NACP + CHX	59.19% ±6.14 ^a^
CHX	58.33% ±6.02 ^a^
Ca(OH)_2_	36.81% ±2.97 ^b^
*p*-value	0.01 × 10^-16^

Mean percentage of dead cells (%) and standard deviation for each experimental group. Different superscript lowercase letters indicate statistically significant differences between groups as determined by one-way ANOVA followed by Tukey’s post hoc test (*p* < 0.05). Groups sharing the same superscript letter are not significantly different

**Table 2:** Pairwise comparisons of dead cell percentages (Tukey’s HSD Post Hoc Test)

Comparison	Mean Difference	Adjusted *p*-value (*p*-adj)	95% Confidence Interval (Lower)	95% Confidence Interval (Upper)	Significant
CHX vs. Ca(OH)₂	-21.815	1.59 × 10^− 11^	-27.50	-16.12	Yes
CHX vs. Control	-51.565	4.88 × 10^− 15^	-57.26	-45.87	Yes
CHX vs. NACP + CHX	0.559	0.993	-5.14	6.25	No
Ca(OH)₂ vs. Control	-29.750	6.99 × 10^− 15^	-35.44	-24.05	Yes
Ca(OH)₂ vs. NACP + CHX	22.374	8.01 × 10^− 12^	16.68	28.06	Yes
Control vs. NACP + CHX	52.124	4.89 × 10^− 15^	46.43	57.81	Yes

*p*-value < 0.05 indicates a statistically significant difference

## Discussion

Enhancing the antimicrobial aspect of endodontic treatment is one of its core research interests [4]. Mechanical shaping is unable to clean the variable anatomical variation of root canal systems, elucidating the importance of irrigants [5, 7]. However, regardless of the irrigation protocol in terms of the irrigants and activation adjuncts, achieving complete disinfection is still impossible [5, 8]. Subsequently, intracanal medicaments are still an important part of endodontics and are frequently called upon [4]. This study evaluates a combination of amorphous calcium phosphate nanoparticles embedded in chlorhexidine in terms of its antibacterial efficiency. This reignited interest in calcium phosphate nanoparticles, especially in cariology and endodontics, stems from its ability to promote remineralization and enhance radicular dentin microhardness [19, 20, 39], especially that calcium phosphate nanoparticles offer an advantage of safety and biocompatibility over traditional nanoparticles (such as nanosilver) which are often associated with undesirable side effects [18]. To the authors’ knowledge, the present study provides the first CLSM-based evaluation of an NACP-loaded CHX intracanal medicament.

The design of this study prioritized clarity of results, especially given its in-vitro nature. The antibacterial effect was assessed using a CLSM, which allows for the observation of live bacteria in root canal walls and dentinal tubules through vital staining, labelled bacterial identification, and 3D visualization of biofilm structural organization [38, 40]. Additionally, this method provides optical sectioning and the ability to study hydrated samples subsurface, which is crucial for analyzing decalcified dentin without surface contaminants or artifacts [40]. Thus CLSM provides spatial visualization and quantification of bacterial viability within the three-dimensional architecture of a mature biofilm. Although complementary methods such as MIC/MBC testing, biomass assays (e.g., Crystal Violet), or molecular techniques such as qPCR are widely used in antimicrobial research, each interrogates a distinct biological endpoint. As highlighted by Bankier et al. [41], assay selection significantly influences interpretation of nanoparticle-based antimicrobial activity. In the present study, the 2% CHX concentration substantially exceeds frequently reported MIC and MBC values for *E. faecalis*; for example, MIC = ~ 6.25 µg/mL and MBC = ~ 50 µg/mL reported in vitro for CHX against ATCC *E. faecalis* strains [42], rendering planktonic susceptibility testing less informative for the specific objective of evaluating biofilm penetration and in situ killing efficacy. Furthermore, biomass assays quantify total organic matter without distinguishing viability, and molecular methods such as qPCR may detect DNA from both live and dead bacteria unless additional viability discrimination techniques are employed [41]. Therefore, CLSM was considered the most appropriate modality for directly assessing the live/dead ratio within the intact biofilm structure.

Also, while the authors are aware that the *E. faecalis* is not considered the primary pathogen of retreatments as once believed [43, 44], this choice was done given its known ability to resist Ca(OH)_2_ and its array of defence mechanisms including aggregation substance, cytolysin, lytic enzymes, lipoteichoic acid, and pheromones [45], not to mention its ability to penetrate deep into dentinal tubules and remain viable for prolonged periods before it reflourishes and develop biofilms [2]. The choice of Ca(OH)_2_ as a main comparator was logical given its status as the most commonly used intracanal medicament [4]. The CHX group was included to assess whether the new NACP + CHX mix could either potentiate or match the efficacy of an equal volume of CHX. If the combination could achieve similar potency, the added benefit of dentin strengthening would justify its use.

In this study, the NACP + CHX group demonstrated the highest mean dead bacterial-cells percentage (59.19%), although without a significant difference from the CHX group (58.33%). However, both groups were significantly more efficient than the Ca(OH)_2_ group (36.81%), which surpassed only the positive control group. Thus, the null hypothesis was rejected. It is worth reporting that a large observed effect size (η² = 0.957) was observed indicating that the present sample size was sufficient to detect substantial intergroup differences. Nevertheless, smaller differences between CHX and NACP–CHX may require larger sample sizes for further discrimination.

Despite being the gold standard of intracanal medicaments, Ca(OH)_2_ demonstrated the least efficiency against *E. faecalis* biofilm amongst the experimental groups. This can be explained by the fact that Ca(OH)_2_ exerts its antimicrobial action through dissociation and release of its hydroxyl ions, which can not penetrate deep enough into dentinal tubules where *E. faecalis* are present [12]. Also, Ca(OH)_2_ fails to maintain a high pH environment due to the buffering effect of dentin [11], not to mention the proton pump activity of *E. faecalis* that allows it to resist higher pH environments [46]. On the other hand, CHX’s broad-spectrum antibacterial action is well documented. This potency is attributed to its interaction with the negatively charged phospholipids and lipopolysaccharides on the cell membrane, thus hijacking the bacterial active and passive transport mechanisms, increasing cell’s permeability, and causing leakage of intracellular components [47]. Leakage of adenosine triphosphate and nucleic acids, plus the coagulation of cytoplasm result in cell death. Also, CHX has the ability to bind to hydroxyapatite changing its electrical field to compete with the binding of bacteria and decreasing their adherence to underlying surfaces. However, the role of such antibacterial mechanism in clinical context is now questionable [5].

The tested mix of NACP + CHX nanoparticles showed comparable antibacterial efficiency when compared to the CHX and significantly higher efficiency than Ca(OH)_2_. It may be hypothesized that calcium ion release could have contributed to the antibacterial activity; however, ion release was not measured in the present study, and this justification remains speculative. Also, it is probable that the negatively charged surface of the nanoparticles coulc cause an electrostatic attraction to the peptidoglycan layer surrounding Gram-positive bacteria [18]. A third postulate is its ability to induce pH fluctuations that are hypothesized to destabilize microorganisms [48], and finally, the smaller particle size and amorphous phase of CP can potentiate the antibacterial activity compared to larger particle sizes and crystalline phases [15–17].

While the NACP + CHX group did not show a statistically significant improvement over CHX alone in terms of bacterial reduction, this outcome should not be interpreted as a lack of clinical value. On the contrary, the comparable antimicrobial efficacy, when coupled with the unique ability of NACP to enhance radicular dentin microhardness [19–22], presents a compelling case for this combination. In scenarios where enhancing dentinal hardness is advantageous (such as regenerative procedures), the dual function of this medicament could offer a meaningful clinical advantage. Thus, even without superior antibacterial performance, the added potential for dentin reinforcement supports its consideration as a viable adjunct, especially that the nanoscale of NACP may enhance its ability to penetrate dentinal tubules compared with conventional CHX formulations. Smaller particle size may facilitate deeper diffusion within tubule architecture [49] and increase contact surface area with biofilm-embedded bacteria [15, 17]. However, tubule penetration was not directly measured in the present study and remains to be investigated.

The two-week incubation period employed in this in-vitro study allowed for the assessment of the medicaments’ maximal antibacterial potential under controlled conditions, however, it is important to acknowledge that this extended contact time may not fully reflect the shorter interappointment intervals often observed in contemporary clinical protocols. Single-visit treatments are increasingly common; however, multi-visit approaches with 1–2 week medicament placement remain a vital strategy for managing complex cases such as persistent infections, presence of sinus tracts, exudation, or symptomatic apical periodontitis [50–52]. Studies have shown that both 7- and 14-day intracanal medication protocols are effective in reducing bacterial load and inflammatory markers, with some evidence suggesting benefits of longer durations for cytokine reduction [50]. Therefore, while our findings demonstrate the inherent efficacy of the medicaments, extrapolation to in-vivo conditions should consider the dynamic clinical environment where contact times can vary. Future research could explore the antibacterial efficacy of these medicaments over shorter, clinically relevant timeframes to further bridge the gap between in-vitro observations and clinical outcomes.

Beyond antibacterial efficacy, the clinical application of CHX-based medicaments involves considerations such as potential dentin discoloration and interactions with sodium hypochlorite, which can form a parachloroaniline (PCA) precipitate [53, 54]. Furthermore, the presence of residual medicament may influence the bond strength of subsequent restorative materials [55], and while the NACP component is intended to enhance dentin properties [20], its specific interaction with adhesive systems remains to be investigated. These factors, along with the optimization of contact time, will be the focus of subsequent translational studies.

This study has limitations that require further research to mitigate. While informative, mono-species models are limited in their ability to fully mimic the clinical microbial environment, as it does not replicate the structural and metabolic complexity of polymicrobial endodontic biofilms [43]. The endodontic microflora is polymicrobial in nature; however, this study employed a single-species biofilm model focusing only on E. faecalis. This choice limits the generalizability of the findings to the broader polymicrobial context observed in clinical scenarios. Also, only single-rooted teeth were used; variations in results may be expected if identical procedures were applied to multi-rooted teeth with their more complex root canal anatomy [6, 56]. Also dentin disc models do not replicate canal anatomy, however, we opted for them because they allow standardized biofilm development and controlled medicament exposure, which is more appropriate for a preliminary exploration. Additionally, dose–response relationships were not investigated, and only a single formulation concentration was evaluated. Finally, although gelatin is not reported to exhibit intrinsic antibacterial activity, variations in carrier composition may influence material diffusion and biofilm interaction. The absence of a vehicle-only control group should therefore be acknowledged as a limitation. Subsequently, additional studies, including multi-species laboratory investigations and animal trials, are necessary to confirm the efficiency and the safety of the combination tested in this study.

## Conclusion

A novel intracanal medication comprised of amorphous calcium phosphate nanoparticles embedded in chlorhexidine has a comparable antibacterial efficacy to CHX and a superior efficacy against *E. faecalis* biofilm when compared to the gold standard Ca(OH)_2_ paste. When considered alongside previously reported improvements in radicular dentin microhardness using the same formulation [20], these findings suggest that the material may represent a promising formulation. Nevertheless, given the preliminary in vitro nature of the present investigation, further studies evaluating multi-species biofilms, cytocompatibility, diffusion characteristics, and in-vivo performance are required before clinical translation can be even considered.

## Supplementary Information

Supplementary Material 1

## Data Availability

The datasets generated and/or analyzed during the current study are not publicly available but are available from the corresponding author on reasonable request.

## Abbreviations

Ca(OH)_2_: Calcium hydroxide
CHX: Chlorhexidine
CLSM: Confocal laser scanning microscope
NACP: Nanoparticles of amorphous calcium phosphate
